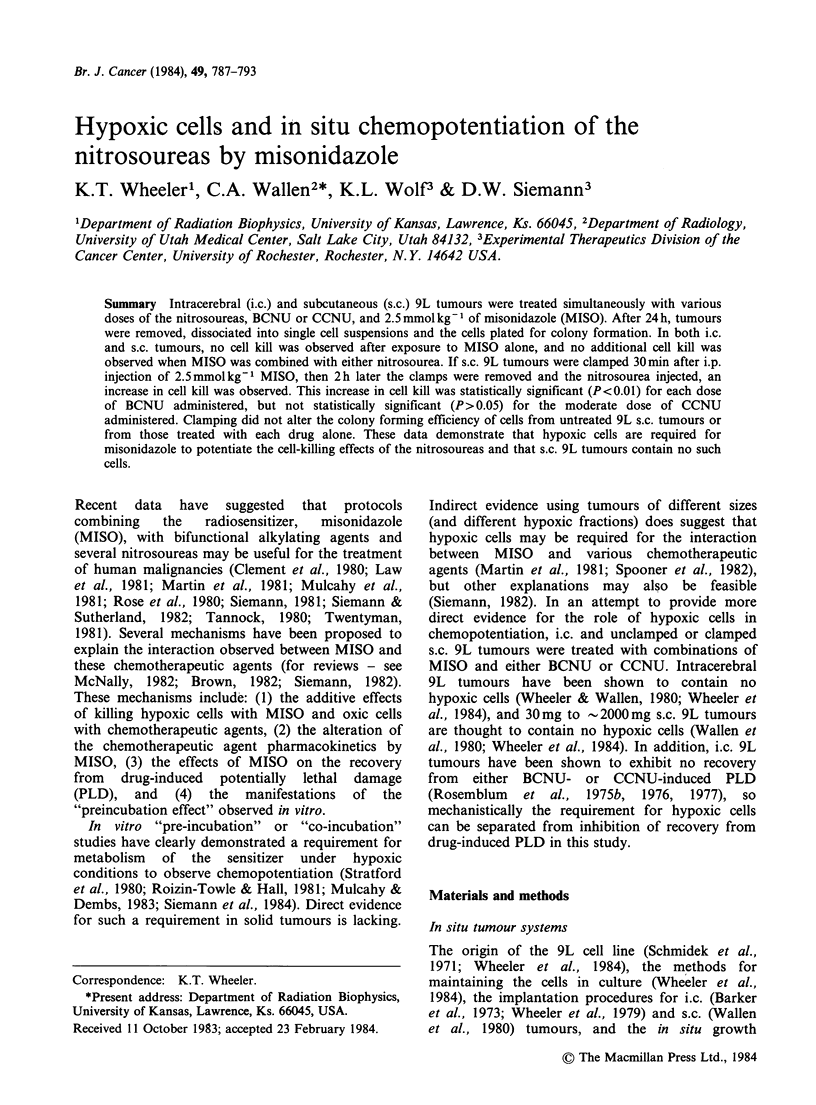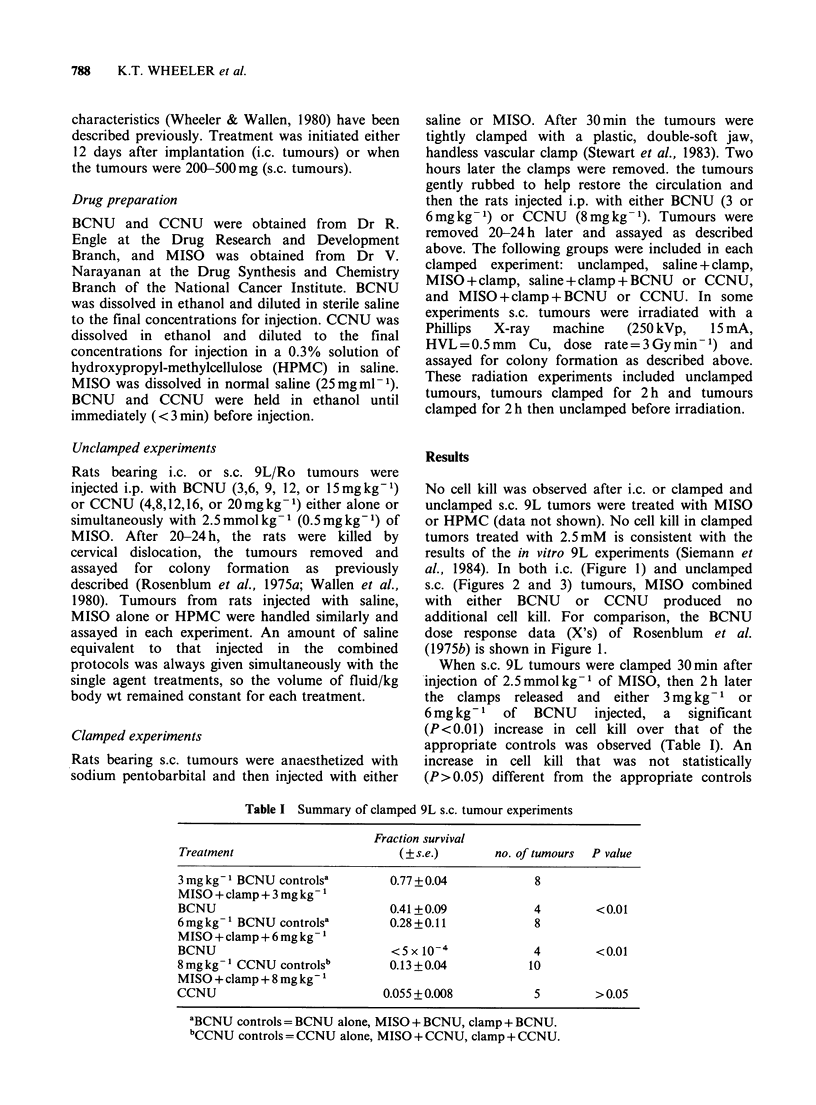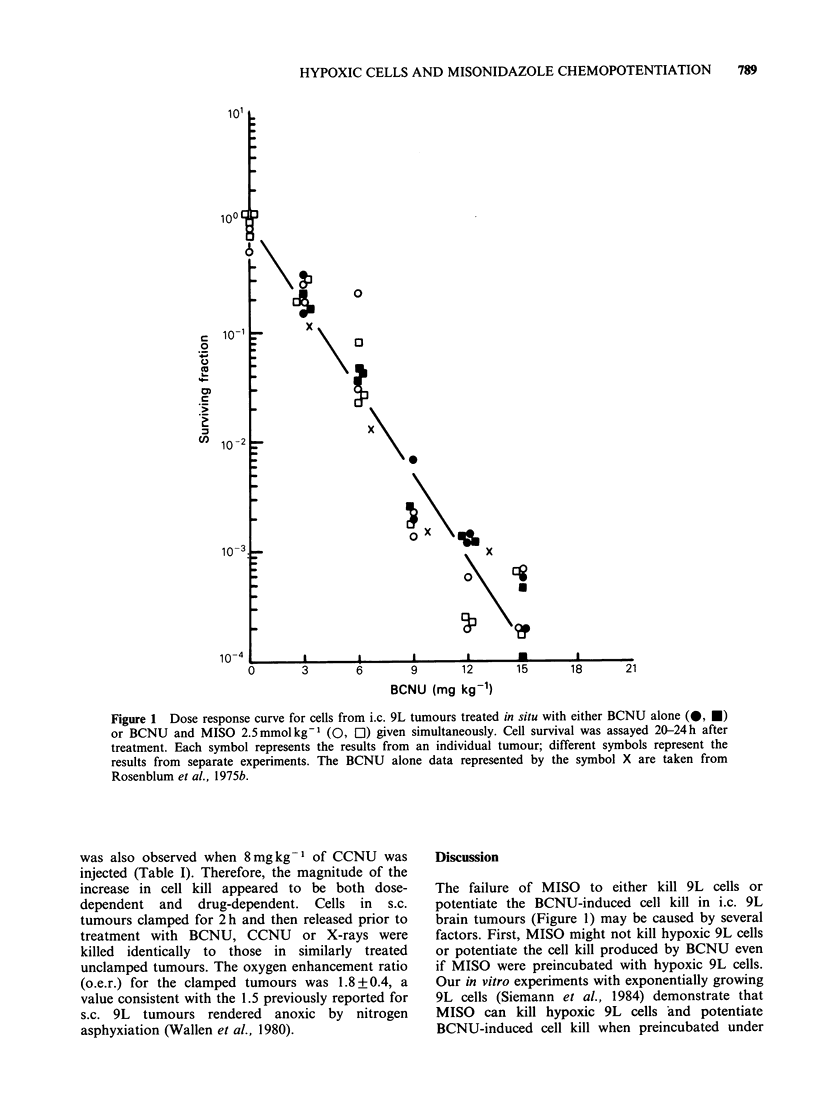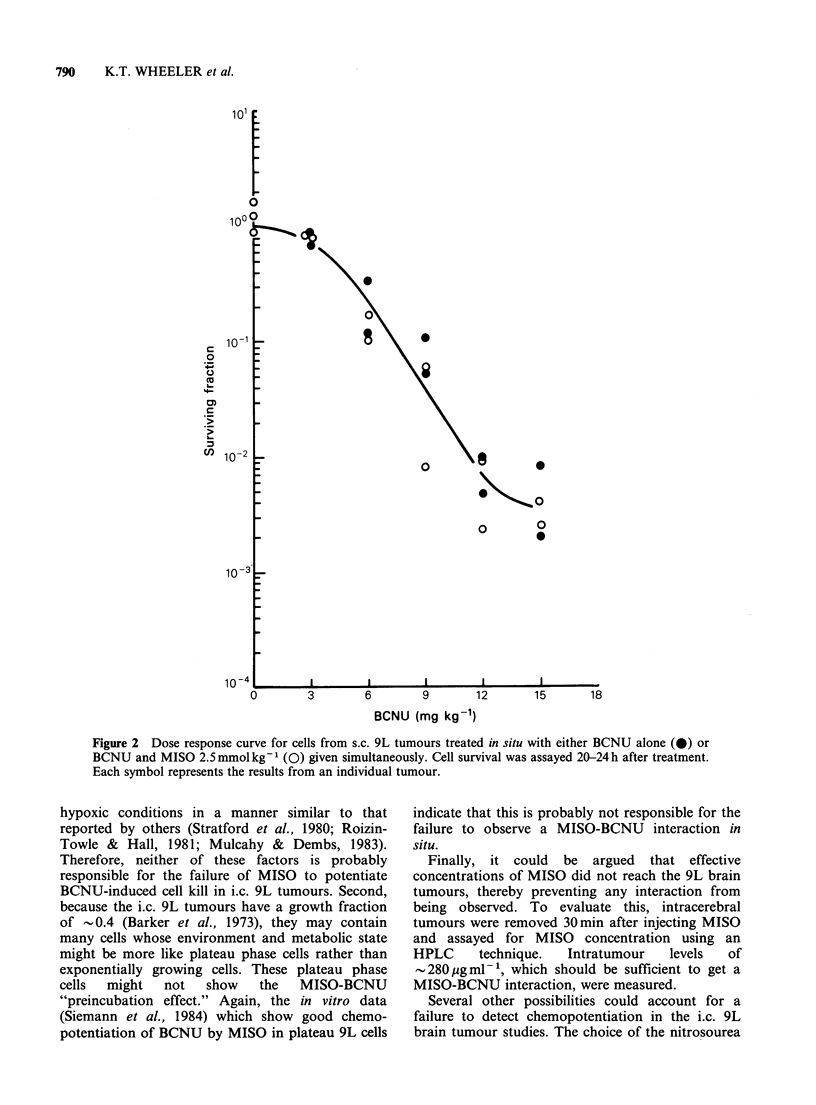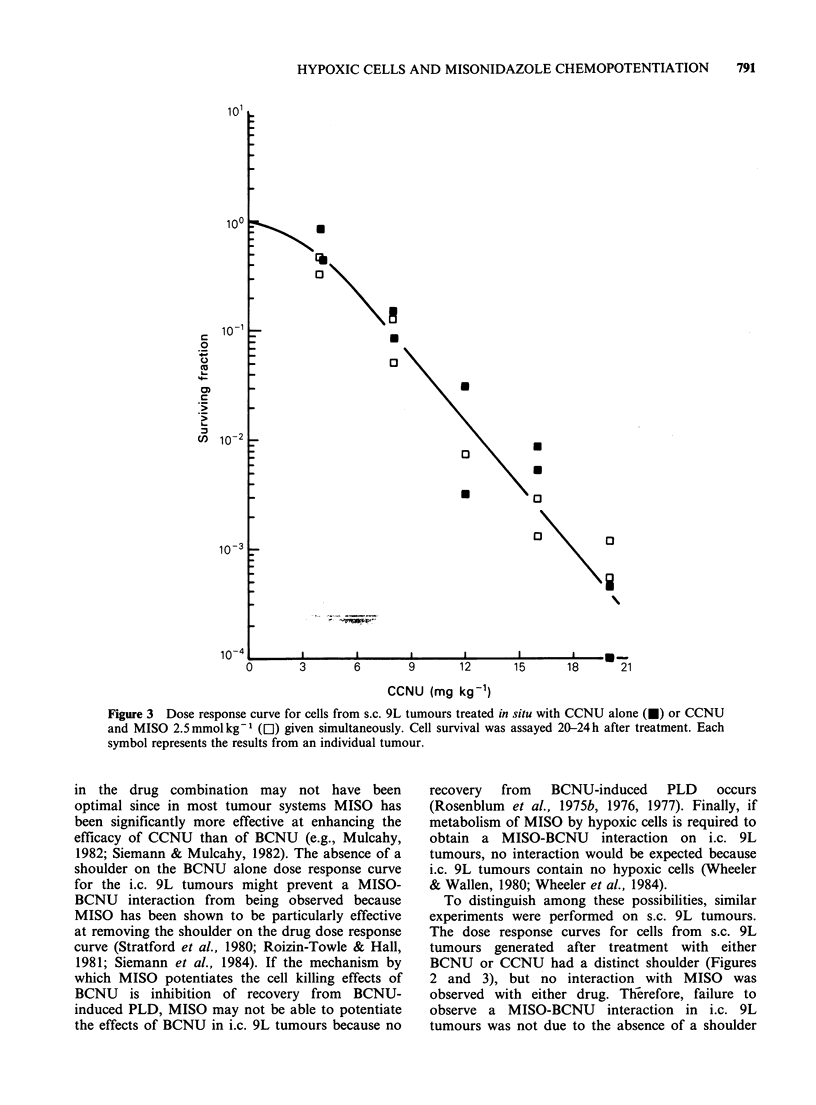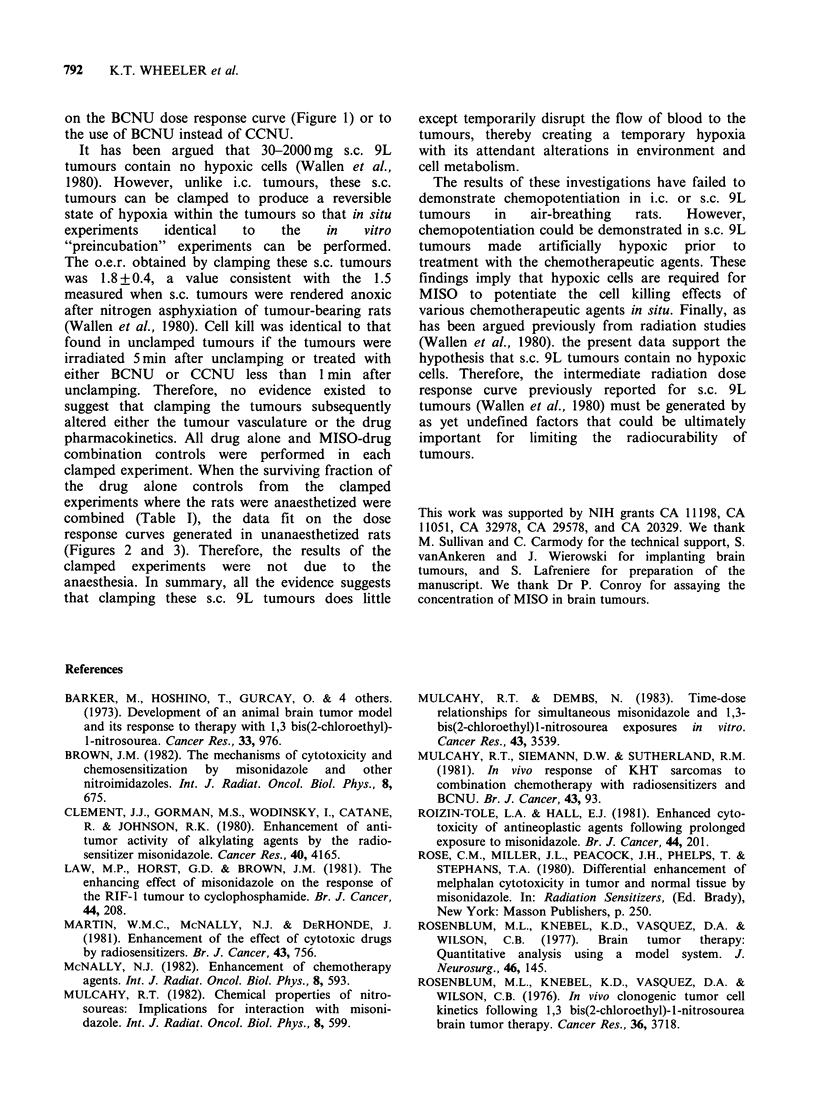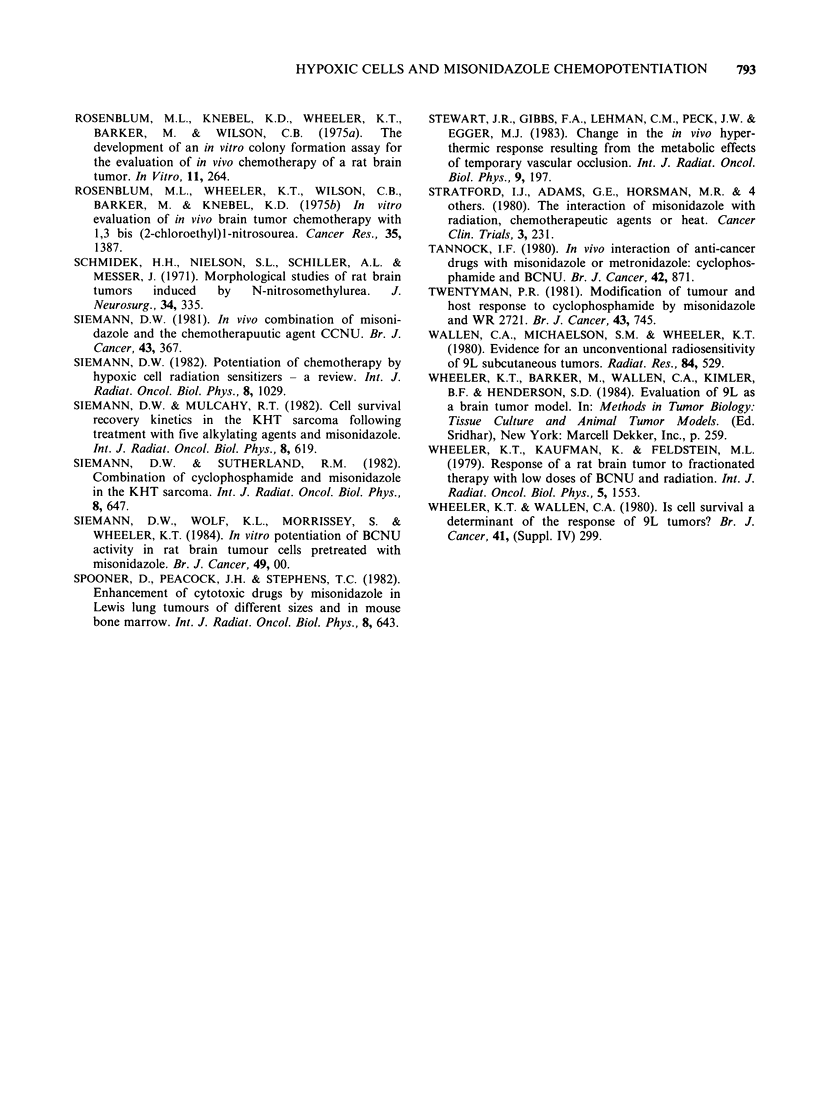# Hypoxic cells and in situ chemopotentiation of the nitrosoureas by misonidazole.

**DOI:** 10.1038/bjc.1984.122

**Published:** 1984-06

**Authors:** K. T. Wheeler, C. A. Wallen, K. L. Wolf, D. W. Siemann

## Abstract

Intracerebral (i.c.) and subcutaneous (s.c.) 9L tumours were treated simultaneously with various doses of the nitrosoureas, BCNU or CCNU, and 2.5 mmol kg-1 of misonidazole (MISO). After 24 h, tumours were removed, dissociated into single cell suspensions and the cells plated for colony formation. In both i.c. and s.c. tumours, no cell kill was observed after exposure to MISO alone, and no additional cell kill was observed when MISO was combined with either nitrosourea. If s.c. 9L tumours were clamped 30 min after i.p. injection of 2.5 mmol kg-1 MISO, then 2 h later the clamps were removed and the nitrosourea injected, an increase in cell kill was observed. This increase in cell kill was statistically significant (P less than 0.01) for each dose of BCNU administered, but not statistically significant (P greater than 0.05) for the moderate dose of CCNU administered. Clamping did not alter the colony forming efficiency of cells from untreated 9L s.c. tumours or from those treated with each drug alone. These data demonstrate that hypoxic cells are required for misonidazole to potentiate the cell-killing effects of the nitrosoureas and that s.c. 9L tumours contain no such cells.


*Br. J. Cancer* (1984), **49**, 787–793

### Hypoxic cells and in situ chemopotentiation of the nitrosoureas by misonidazole

K.T. Wheeler[1], C.A. Wallen[2]*, K.L. Wolf[3] & D.W. Siemann[3]

[1]*Department of Radiation Biophysics, University of Kansas, Lawrence, Ks. 66045,* [2]*Department of Radiology, University of Utah Medical Center, Salt Lake City, Utah 84132,* [3]*Experimental Therapeutics Division of the Cancer Center, University of Rochester, Rochester, N.Y. 14642 USA.*

**Summary** Intracerebral (i.c.) and subcutaneous (s.c.) 9L tumours were treated simultaneously with various doses of the nitrosoureas, BCNU or CCNU, and 2.5 mmol kg$^{-1}$ of misonidazole (MISO). After 24 h, tumours were removed, dissociated into single cell suspensions and the cells plated for colony formation. In both i.c. and s.c. tumours, no cell kill was observed after exposure to MISO alone, and no additional cell kill was observed when MISO was combined with either nitrosourea. If s.c. 9L tumours were clamped 30 min after i.p. injection of 2.5 mmol kg$^{-1}$ MISO, then 2 h later the clamps were removed and the nitrosourea injected, an increase in cell kill was observed. This increase in cell kill was statistically significant ($P<0.01$) for each dose of BCNU administered, but not statistically significant ($P>0.05$) for the moderate dose of CCNU administered. Clamping did not alter the colony forming efficiency of cells from untreated 9L s.c. tumours or from those treated with each drug alone. These data demonstrate that hypoxic cells are required for misonidazole to potentiate the cell-killing effects of the nitrosoureas and that s.c. 9L tumours contain no such cells.

Recent data have suggested that protocols combining the radiosensitizer, misonidazole (MISO), with bifunctional alkylating agents and several nitrosoureas may be useful for the treatment of human malignancies (Clement *et al.*, 1980; Law *et al.*, 1981; Martin *et al.*, 1981; Mulcahy *et al.*, 1981; Rose *et al.*, 1980; Siemann, 1981; Siemann & Sutherland, 1982; Tannock, 1980; Twentyman, 1981). Several mechanisms have been proposed to explain the interaction observed between MISO and these chemotherapeutic agents (for reviews – see McNally, 1982; Brown, 1982; Siemann, 1982). These mechanisms include: (1) the additive effects of killing hypoxic cells with MISO and oxic cells with chemotherapeutic agents, (2) the alteration of the chemotherapeutic agent pharmacokinetics by MISO, (3) the effects of MISO on the recovery from drug-induced potentially lethal damage (PLD), and (4) the manifestations of the "preincubation effect" observed *in vitro*.

*In vitro* "pre-incubation" or "co-incubation" studies have clearly demonstrated a requirement for metabolism of the sensitizer under hypoxic conditions to observe chemopotentiation (Stratford *et al.*, 1980; Roizin-Towle & Hall, 1981; Mulcahy & Dembs, 1983; Siemann *et al.*, 1984). Direct evidence for such a requirement in solid tumours is lacking. Indirect evidence using tumours of different sizes (and different hypoxic fractions) does suggest that hypoxic cells may be required for the interaction between MISO and various chemotherapeutic agents (Martin *et al.*, 1981; Spooner *et al.*, 1982), but other explanations may also be feasible (Siemann, 1982). In an attempt to provide more direct evidence for the role of hypoxic cells in chemopotentiation, i.c. and unclamped or clamped s.c. 9L tumours were treated with combinations of MISO and either BCNU or CCNU. Intracerebral 9L tumours have been shown to contain no hypoxic cells (Wheeler & Wallen, 1980; Wheeler *et al.*, 1984), and 30 mg to ~2000 mg s.c. 9L tumours are thought to contain no hypoxic cells (Wallen *et al.*, 1980; Wheeler *et al.*, 1984). In addition, i.c. 9L tumours have been shown to exhibit no recovery from either BCNU- or CCNU-induced PLD (Rosemblum *et al.*, 1975*b*, 1976, 1977), so mechanistically the requirement for hypoxic cells can be separated from inhibition of recovery from drug-induced PLD in this study.

### Materials and methods

### *In situ tumour systems*

The origin of the 9L cell line (Schmidek *et al.*, 1971; Wheeler *et al.*, 1984), the methods for maintaining the cells in culture (Wheeler *et al.*, 1984), the implantation procedures for i.c. (Barker *et al.*, 1973; Wheeler *et al.*, 1979) and s.c. (Wallen *et al.*, 1980) tumours, and the *in situ* growth

Correspondence: K.T. Wheeler.

*Present address: Department of Radiation Biophysics, University of Kansas, Lawrence, Ks. 66045, USA.

Received 11 October 1983; accepted 23 February 1984.

© The Macmillan Press Ltd., 1984

characteristics (Wheeler & Wallen, 1980) have been described previously. Treatment was initiated either 12 days after implantation (i.c. tumours) or when the tumours were 200–500 mg (s.c. tumours).

*Drug preparation*

BCNU and CCNU were obtained from Dr R. Engle at the Drug Research and Development Branch, and MISO was obtained from Dr V. Narayanan at the Drug Synthesis and Chemistry Branch of the National Cancer Institute. BCNU was dissolved in ethanol and diluted in sterile saline to the final concentrations for injection. CCNU was dissolved in ethanol and diluted to the final concentrations for injection in a 0.3% solution of hydroxypropyl-methylcellulose (HPMC) in saline. MISO was dissolved in normal saline (25 mg ml$^{-1}$). BCNU and CCNU were held in ethanol until immediately (<3 min) before injection.

*Unclamped experiments*

Rats bearing i.c. or s.c. 9L/Ro tumours were injected i.p. with BCNU (3,6, 9, 12, or 15 mg kg$^{-1}$) or CCNU (4,8,12,16, or 20 mg kg$^{-1}$) either alone or simultaneously with 2.5 mmol kg$^{-1}$ (0.5 mg kg$^{-1}$) of MISO. After 20–24 h, the rats were killed by cervical dislocation, the tumours removed and assayed for colony formation as previously described (Rosenblum *et al.*, 1975*a*; Wallen *et al.*, 1980). Tumours from rats injected with saline, MISO alone or HPMC were handled similarly and assayed in each experiment. An amount of saline equivalent to that injected in the combined protocols was always given simultaneously with the single agent treatments, so the volume of fluid/kg body wt remained constant for each treatment.

*Clamped experiments*

Rats bearing s.c. tumours were anaesthetized with sodium pentobarbital and then injected with either saline or MISO. After 30 min the tumours were tightly clamped with a plastic, double-soft jaw, handless vascular clamp (Stewart *et al.*, 1983). Two hours later the clamps were removed. the tumours gently rubbed to help restore the circulation and then the rats injected i.p. with either BCNU (3 or 6 mg kg$^{-1}$) or CCNU (8 mg kg$^{-1}$). Tumours were removed 20–24 h later and assayed as described above. The following groups were included in each clamped experiment: unclamped, saline + clamp, MISO + clamp, saline + clamp + BCNU or CCNU, and MISO + clamp + BCNU or CCNU. In some experiments s.c. tumours were irradiated with a Phillips X-ray machine (250 kVp, 15 mA, HVL = 0.5 mm Cu, dose rate = 3 Gy min$^{-1}$) and assayed for colony formation as described above. These radiation experiments included unclamped tumours, tumours clamped for 2 h and tumours clamped for 2 h then unclamped before irradiation.

### Results

No cell kill was observed after i.c. or clamped and unclamped s.c. 9L tumors were treated with MISO or HPMC (data not shown). No cell kill in clamped tumors treated with 2.5 mM is consistent with the results of the *in vitro* 9L experiments (Siemann *et al.*, 1984). In both i.c. (Figure 1) and unclamped s.c. (Figures 2 and 3) tumours, MISO combined with either BCNU or CCNU produced no additional cell kill. For comparison, the BCNU dose response data (X's) of Rosenblum *et al.* (1975*b*) is shown in Figure 1.

When s.c. 9L tumours were clamped 30 min after injection of 2.5 mmol kg$^{-1}$ of MISO, then 2 h later the clamps released and either 3 mg kg$^{-1}$ or 6 mg kg$^{-1}$ of BCNU injected, a significant ($P<0.01$) increase in cell kill over that of the appropriate controls was observed (Table I). An increase in cell kill that was not statistically ($P>0.05$) different from the appropriate controls

**Table I** Summary of clamped 9L s.c. tumour experiments

| *Treatment* | *Fraction survival* (±*s.e.*) | *no. of tumours* | *P value* |
|---|---|---|---|
| 3 mg kg$^{-1}$ BCNU controls[a] | 0.77 ± 0.04 | 8 | |
| MISO + clamp + 3 mg kg$^{-1}$ BCNU | 0.41 ± 0.09 | 4 | <0.01 |
| 6 mg kg$^{-1}$ BCNU controls[a] | 0.28 ± 0.11 | 8 | |
| MISO + clamp + 6 mg kg$^{-1}$ BCNU | $<5\times10^{-4}$ | 4 | <0.01 |
| 8 mg kg$^{-1}$ CCNU controls[b] | 0.13 ± 0.04 | 10 | |
| MISO + clamp + 8 mg kg$^{-1}$ CCNU | 0.055 ± 0.008 | 5 | >0.05 |

[a]BCNU controls = BCNU alone, MISO + BCNU, clamp + BCNU.
[b]CCNU controls = CCNU alone, MISO + CCNU, clamp + CCNU.

**Figure 1** Dose response curve for cells from i.c. 9L tumours treated *in situ* with either BCNU alone (●, ■) or BCNU and MISO 2.5 mmol kg$^{-1}$ (○, □) given simultaneously. Cell survival was assayed 20–24 h after treatment. Each symbol represents the results from an individual tumour; different symbols represent the results from separate experiments. The BCNU alone data represented by the symbol X are taken from Rosenblum *et al.*, 1975*b*.

was also observed when 8 mg kg$^{-1}$ of CCNU was injected (Table I). Therefore, the magnitude of the increase in cell kill appeared to be both dose-dependent and drug-dependent. Cells in s.c. tumours clamped for 2 h and then released prior to treatment with BCNU, CCNU or X-rays were killed identically to those in similarly treated unclamped tumours. The oxygen enhancement ratio (o.e.r.) for the clamped tumours was $1.8 \pm 0.4$, a value consistent with the 1.5 previously reported for s.c. 9L tumours rendered anoxic by nitrogen asphyxiation (Wallen *et al.*, 1980).

### Discussion

The failure of MISO to either kill 9L cells or potentiate the BCNU-induced cell kill in i.c. 9L brain tumours (Figure 1) may be caused by several factors. First, MISO might not kill hypoxic 9L cells or potentiate the cell kill produced by BCNU even if MISO were preincubated with hypoxic 9L cells. Our *in vitro* experiments with exponentially growing 9L cells (Siemann *et al.*, 1984) demonstrate that MISO can kill hypoxic 9L cells and potentiate BCNU-induced cell kill when preincubated under

**Figure 2** Dose response curve for cells from s.c. 9L tumours treated *in situ* with either BCNU alone (●) or BCNU and MISO 2.5 mmol kg$^{-1}$ (○) given simultaneously. Cell survival was assayed 20–24 h after treatment. Each symbol represents the results from an individual tumour.

hypoxic conditions in a manner similar to that reported by others (Stratford *et al.*, 1980; Roizin-Towle & Hall, 1981; Mulcahy & Dembs, 1983). Therefore, neither of these factors is probably responsible for the failure of MISO to potentiate BCNU-induced cell kill in i.c. 9L tumours. Second, because the i.c. 9L tumours have a growth fraction of ~0.4 (Barker *et al.*, 1973), they may contain many cells whose environment and metabolic state might be more like plateau phase cells rather than exponentially growing cells. These plateau phase cells might not show the MISO-BCNU "preincubation effect." Again, the *in vitro* data (Siemann *et al.*, 1984) which show good chemopotentiation of BCNU by MISO in plateau 9L cells indicate that this is probably not responsible for the failure to observe a MISO-BCNU interaction *in situ*.

Finally, it could be argued that effective concentrations of MISO did not reach the 9L brain tumours, thereby preventing any interaction from being observed. To evaluate this, intracerebral tumours were removed 30 min after injecting MISO and assayed for MISO concentration using an HPLC technique. Intratumour levels of ~280 $\mu$g ml$^{-1}$, which should be sufficient to get a MISO-BCNU interaction, were measured.

Several other possibilities could account for a failure to detect chemopotentiation in the i.c. 9L brain tumour studies. The choice of the nitrosourea

**Figure 3** Dose response curve for cells from s.c. 9L tumours treated *in situ* with CCNU alone (■) or CCNU and MISO 2.5 mmol kg$^{-1}$ (□) given simultaneously. Cell survival was assayed 20–24 h after treatment. Each symbol represents the results from an individual tumour.

in the drug combination may not have been optimal since in most tumour systems MISO has been significantly more effective at enhancing the efficacy of CCNU than of BCNU (e.g., Mulcahy, 1982; Siemann & Mulcahy, 1982). The absence of a shoulder on the BCNU alone dose response curve for the i.c. 9L tumours might prevent a MISO-BCNU interaction from being observed because MISO has been shown to be particularly effective at removing the shoulder on the drug dose response curve (Stratford *et al.*, 1980; Roizin-Towle & Hall, 1981; Siemann *et al.*, 1984). If the mechanism by which MISO potentiates the cell killing effects of BCNU is inhibition of recovery from BCNU-induced PLD, MISO may not be able to potentiate the effects of BCNU in i.c. 9L tumours because no recovery from BCNU-induced PLD occurs (Rosenblum *et al.*, 1975*b*, 1976, 1977). Finally, if metabolism of MISO by hypoxic cells is required to obtain a MISO-BCNU interaction on i.c. 9L tumours, no interaction would be expected because i.c. 9L tumours contain no hypoxic cells (Wheeler & Wallen, 1980; Wheeler *et al.*, 1984).

To distinguish among these possibilities, similar experiments were performed on s.c. 9L tumours. The dose response curves for cells from s.c. 9L tumours generated after treatment with either BCNU or CCNU had a distinct shoulder (Figures 2 and 3), but no interaction with MISO was observed with either drug. Therefore, failure to observe a MISO-BCNU interaction in i.c. 9L tumours was not due to the absence of a shoulder on the BCNU dose response curve (Figure 1) or to the use of BCNU instead of CCNU.

It has been argued that 30–2000 mg s.c. 9L tumours contain no hypoxic cells (Wallen *et al.*, 1980). However, unlike i.c. tumours, these s.c. tumours can be clamped to produce a reversible state of hypoxia within the tumours so that *in situ* experiments identical to the *in vitro* "preincubation" experiments can be performed. The o.e.r. obtained by clamping these s.c. tumours was $1.8 \pm 0.4$, a value consistent with the 1.5 measured when s.c. tumours were rendered anoxic after nitrogen asphyxiation of tumour-bearing rats (Wallen *et al.*, 1980). Cell kill was identical to that found in unclamped tumours if the tumours were irradiated 5 min after unclamping or treated with either BCNU or CCNU less than 1 min after unclamping. Therefore, no evidence existed to suggest that clamping the tumours subsequently altered either the tumour vasculature or the drug pharmacokinetics. All drug alone and MISO-drug combination controls were performed in each clamped experiment. When the surviving fraction of the drug alone controls from the clamped experiments where the rats were anaesthetized were combined (Table I), the data fit on the dose response curves generated in unanaesthetized rats (Figures 2 and 3). Therefore, the results of the clamped experiments were not due to the anaesthesia. In summary, all the evidence suggests that clamping these s.c. 9L tumours does little except temporarily disrupt the flow of blood to the tumours, thereby creating a temporary hypoxia with its attendant alterations in environment and cell metabolism.

The results of these investigations have failed to demonstrate chemopotentiation in i.c. or s.c. 9L tumours in air-breathing rats. However, chemopotentiation could be demonstrated in s.c. 9L tumours made artificially hypoxic prior to treatment with the chemotherapeutic agents. These findings imply that hypoxic cells are required for MISO to potentiate the cell killing effects of various chemotherapeutic agents *in situ*. Finally, as has been argued previously from radiation studies (Wallen *et al.*, 1980). the present data support the hypothesis that s.c. 9L tumours contain no hypoxic cells. Therefore, the intermediate radiation dose response curve previously reported for s.c. 9L tumours (Wallen *et al.*, 1980) must be generated by as yet undefined factors that could be ultimately important for limiting the radiocurability of tumours.

This work was supported by NIH grants CA 11198, CA 11051, CA 32978, CA 29578, and CA 20329. We thank M. Sullivan and C. Carmody for the technical support, S. vanAnkeren and J. Wierowski for implanting brain tumours, and S. Lafreniere for preparation of the manuscript. We thank Dr P. Conroy for assaying the concentration of MISO in brain tumours.

### References

BARKER, M., HOSHINO, T., GURCAY, O. & 4 others. (1973). Development of an animal brain tumor model and its response to therapy with 1,3 bis(2-chloroethyl)-1-nitrosourea. *Cancer Res.*, **33**, 976.

BROWN, J.M. (1982). The mechanisms of cytotoxicity and chemosensitization by misonidazole and other nitroimidazoles. *Int. J. Radiat. Oncol. Biol. Phys.*, **8**, 675.

CLEMENT, J.J., GORMAN, M.S., WODINSKY, I., CATANE, R. & JOHNSON, R.K. (1980). Enhancement of antitumor activity of alkylating agents by the radiosensitizer misonidazole. *Cancer Res.*, **40**, 4165.

LAW, M.P., HORST, G.D. & BROWN, J.M. (1981). The enhancing effect of misonidazole on the response of the RIF-1 tumour to cyclophosphamide. *Br. J. Cancer*, **44**, 208.

MARTIN, W.M.C., McNALLY, N.J. & DeRHONDE, J. (1981). Enhancement of the effect of cytotoxic drugs by radiosensitizers. *Br. J. Cancer*, **43**, 756.

McNALLY, N.J. (1982). Enhancement of chemotherapy agents. *Int. J. Radiat. Oncol. Biol. Phys.*, **8**, 593.

MULCAHY, R.T. (1982). Chemical properties of nitrosoureas: Implications for interaction with misonidazole. *Int. J. Radiat. Oncol. Biol. Phys.*, **8**, 599.

MULCAHY, R.T. & DEMBS, N. (1983). Time-dose relationships for simultaneous misonidazole and 1,3-bis(2-chloroethyl)1-nitrosourea exposures *in vitro*. *Cancer Res.*, **43**, 3539.

MULCAHY, R.T., SIEMANN, D.W. & SUTHERLAND, R.M. (1981). *In vivo* response of KHT sarcomas to combination chemotherapy with radiosensitizers and BCNU. *Br. J. Cancer*, **43**, 93.

ROIZIN-TOLE, L.A. & HALL, E.J. (1981). Enhanced cytotoxicity of antineoplastic agents following prolonged exposure to misonidazole. *Br. J. Cancer*, **44**, 201.

ROSE, C.M., MILLER, J.L., PEACOCK, J.H., PHELPS, T. & STEPHANS, T.A. (1980). Differential enhancement of melphalan cytotoxicity in tumor and normal tissue by misonidazole. In: *Radiation Sensitizers*, (Ed. Brady), New York: Masson Publishers, p. 250.

ROSENBLUM, M.L., KNEBEL, K.D., VASQUEZ, D.A. & WILSON, C.B. (1977). Brain tumor therapy: Quantitative analysis using a model system. *J. Neurosurg.*, **46**, 145.

ROSENBLUM, M.L., KNEBEL, K.D., VASQUEZ, D.A. & WILSON, C.B. (1976). *In vivo* clonogenic tumor cell kinetics following 1,3 bis(2-chloroethyl)-1-nitrosourea brain tumor therapy. *Cancer Res.*, **36**, 3718.

ROSENBLUM, M.L., KNEBEL, K.D., WHEELER, K.T., BARKER, M. & WILSON, C.B. (1975*a*). The development of an *in vitro* colony formation assay for the evaluation of *in vivo* chemotherapy of a rat brain tumor. *In Vitro*, **11**, 264.

ROSENBLUM, M.L., WHEELER, K.T., WILSON, C.B., BARKER, M. & KNEBEL, K.D. (1975*b*) *In vitro* evaluation of *in vivo* brain tumor chemotherapy with 1,3 bis (2-chloroethyl)1-nitrosourea. *Cancer Res.*, **35**, 1387.

SCHMIDEK, H.H., NIELSON, S.L., SCHILLER, A.L. & MESSER, J. (1971). Morphological studies of rat brain tumors induced by N-nitrosomethylurea. *J. Neurosurg.*, **34**, 335.

SIEMANN, D.W. (1981). *In vivo* combination of misonidazole and the chemotherapuutic agent CCNU. *Br. J. Cancer*, **43**, 367.

SIEMANN, D.W. (1982). Potentiation of chemotherapy by hypoxic cell radiation sensitizers – a review. *Int. J. Radiat. Oncol. Biol. Phys.*, **8**, 1029.

SIEMANN, D.W. & MULCAHY, R.T. (1982). Cell survival recovery kinetics in the KHT sarcoma following treatment with five alkylating agents and misonidazole. *Int. J. Radiat. Oncol. Biol. Phys.*, **8**, 619.

SIEMANN, D.W. & SUTHERLAND, R.M. (1982). Combination of cyclophosphamide and misonidazole in the KHT sarcoma. *Int. J. Radiat. Oncol. Biol. Phys.*, **8**, 647.

SIEMANN, D.W., WOLF, K.L., MORRISSEY, S. & WHEELER, K.T. (1984). *In vitro* potentiation of BCNU activity in rat brain tumour cells pretreated with misonidazole. *Br. J. Cancer*, **49**, 00.

SPOONER, D., PEACOCK, J.H. & STEPHENS, T.C. (1982). Enhancement of cytotoxic drugs by misonidazole in Lewis lung tumours of different sizes and in mouse bone marrow. *Int. J. Radiat. Oncol. Biol. Phys.*, **8**, 643.

STEWART, J.R., GIBBS, F.A., LEHMAN, C.M., PECK, J.W. & EGGER, M.J. (1983). Change in the *in vivo* hyperthermic response resulting from the metabolic effects of temporary vascular occlusion. *Int. J. Radiat. Oncol. Biol. Phys.*, **9**, 197.

STRATFORD, I.J., ADAMS, G.E., HORSMAN, M.R. & 4 others. (1980). The interaction of misonidazole with radiation, chemotherapeutic agents or heat. *Cancer Clin. Trials*, **3**, 231.

TANNOCK, I.F. (1980). *In vivo* interaction of anti-cancer drugs with misonidazole or metronidazole: cyclophosphamide and BCNU. *Br. J. Cancer*, **42**, 871.

TWENTYMAN, P.R. (1981). Modification of tumour and host response to cyclophosphamide by misonidazole and WR 2721. *Br. J. Cancer*, **43**, 745.

WALLEN, C.A., MICHAELSON, S.M. & WHEELER, K.T. (1980). Evidence for an unconventional radiosensitivity of 9L subcutaneous tumors. *Radiat. Res.*, **84**, 529.

WHEELER, K.T., BARKER, M., WALLEN, C.A., KIMLER, B.F. & HENDERSON, S.D. (1984). Evaluation of 9L as a brain tumor model. In: *Methods in Tumor Biology: Tissue Culture and Animal Tumor Models.* (Ed. Sridhar), New York: Marcell Dekker, Inc., p. 259.

WHEELER, K.T., KAUFMAN, K. & FELDSTEIN, M.L. (1979). Response of a rat brain tumor to fractionated therapy with low doses of BCNU and radiation. *Int. J. Radiat. Oncol. Biol. Phys.*, **5**, 1553.

WHEELER, K.T. & WALLEN, C.A. (1980). Is cell survival a determinant of the response of 9L tumors? *Br. J. Cancer*, **41**, (Suppl. IV) 299.